# BIGL: Biochemically Intuitive Generalized Loewe null model for prediction of the expected combined effect compatible with partial agonism and antagonism

**DOI:** 10.1038/s41598-017-18068-5

**Published:** 2017-12-20

**Authors:** Koen Van der Borght, Annelies Tourny, Rytis Bagdziunas, Olivier Thas, Maxim Nazarov, Heather Turner, Bie Verbist, Hugo Ceulemans

**Affiliations:** 1 Discovery Sciences, Janssen Research & Development, Turnhoutseweg 30, B-2340 Beerse, Belgium; 2 Department of Mathematical Modeling, Statistics and Bioinformatics, Ghent University, Coupure Links 653, B-900 Gent, Belgium; 3 Open Analytics, Jupiterstraat 20, B-2600 Antwerpen, Belgium; 4 National Institute for Applied Statistics Research Australia (NIASRA), University of Wollongong, Northfields Avenue, Wollongong, NSW 2522 Australia

## Abstract

Clinical efficacy regularly requires the combination of drugs. For an early estimation of the clinical value of (potentially many) combinations of pharmacologic compounds during discovery, the observed combination effect is typically compared to that expected under a null model. Mechanistic accuracy of that null model is not aspired to; to the contrary, combinations that deviate favorably from the model (and thereby disprove its accuracy) are prioritized. Arguably the most popular null model is the Loewe Additivity model, which conceptually maps any assay under study to a (virtual) single-step enzymatic reaction. It is easy-to-interpret and requires no other information than the concentration-response curves of the individual compounds. However, the original Loewe model cannot accommodate concentration-response curves with different maximal responses and, by consequence, combinations of an agonist with a partial or inverse agonist. We propose an extension, named Biochemically Intuitive Generalized Loewe (BIGL), that can address different maximal responses, while preserving the biochemical underpinning and interpretability of the original Loewe model. In addition, we formulate statistical tests for detecting synergy and antagonism, which allow for detecting statistically significant greater/lesser observed combined effects than expected from the null model. Finally, we demonstrate the novel method through application to several publicly available datasets.

## Introduction

The treatment of heterogeneous disorders such as cancer, infectious diseases and autoimmune diseases is often complicated by adverse effects that limit the tolerable dose of a single agent, or by the gradual development of resistance. Combination therapy, i.e. the simultaneous treatment with multiple therapeutic agents, presents an approach to mitigate such complications. Individual agents are selected that differ in terms of adverse activities and hence can be administered at tolerable doses, yet that all converge on a key disease-causing process, thus maximizing the treatment’s efficacy. A combination of multiple effective drugs also encumbers the evolution of resistance, which then requires the simultaneous evasion of multiple mechanisms of action on the same process. Therefore, combination therapies are increasingly adopted as the standard of care for various diseases^1^.

Consequently, combinations of pharmacological compounds are routinely tested and their activities assessed during early drug discovery. Generating all the data needed for complete mechanistic understanding and modeling of these compound interactions with differential equations is labor intensive and generally not feasible or too costly at this early stage. However, reading out activities in concentration-response, e.g. from a serial dilution of the single agents in the combinations, is typically straightforward and affordable. Conveniently, these single agent results enable the formulation of a plausible expectation of their combined effect under a null model. Comparison of the observed effect of a combination of compounds to that predicted by the null model, then allows an assessment of the potential of that combination. The most promising combinations are the ones with an observed effect that significantly exceeds the expected effect; these are called synergistic.

Notably, the null model does not aim to be mechanistically accurate. In fact, typically the most attractive combinations are the ones that show the largest favorable deviation from the null model. By its very definition, a synergy call rejects the null model, i.e. it disproves the mechanism underlying it. The costly and labor-intensive modelling of the actual mechanism underlying the combination effect of interest is reserved for those few combinations that are the most attractive and that have their favorable effect confirmed. It often involves elaborating systems of differential equations and custom data generation.

The choice of a null model for the early (and scalable) evaluation of compound combinations is mostly inspired by convenience. Can base-line effect expectations be computed leveraging only the limited information that is routinely available during early discovery, i.e. on concentration response curves?

Even though several null models exist, such as Bliss Independence^2^, arguably the most frequently used null model is the one proposed by Loewe^3^. The Loewe model, also known as Concentration Additivity, is a mechanistically inspired and biochemically interpretable model that conceptualizes the assay under study as a (virtual) single-step enzymatic reaction, even if the underlying biology is in fact much more complicated (for instance if the assay relies on entire cells or even organisms), and hence the model is obviously incomplete at best. The conceptual biochemical mechanism posits that the effective binding of an enzyme molecule by any compound switches its activity state (turning it on or off, depending on the activity state of unbound enzyme). Moreover, compound binding to an enzyme molecule is mutually exclusive, i.e. compounds compete for binding. Under this model (under the two classical Loewe constraints), the fraction of bound and thereby identically affected enzyme molecules increases with the concentration of any compound. Once all enzyme molecules are occupied by compound, a maximal response is achieved which is inherently the same for all effective compounds and their combination. However, the common maximal response implied by the classical Loewe model regularly proves untenable. Thus, in cellular assays it occurs that different compounds elicit maximal responses in the same assay that differ in magnitude (partial agonism) or even in direction (agonism/antagonism, for instance in receptor signaling). The classical Loewe model cannot accommodate these cases.

Although several methods have been described that can cope with partial agonism or agonism/antagonism, these are no longer easily biochemically interpretable (see Discussion). Therefore, we propose a novel direct generalization of the Loewe model, called BIGL (Biochemically Intuitive Generalized Loewe), which maintains the biochemical interpretability of the classical Loewe model, even in cases of partial agonism and antagonism.

The BIGL model together with two bootstrap statistical tests for synergy calling (MeanR/MaxR) have been implemented in R as the R package *BIGL* (https://cran.r-project.org/web/packages/BIGL). We demonstrate the method on some publicly available datasets.

## Results

### Hill equation: occupancy or readout as a function of concentration

The Hill equation is the established model to describe the concentration-response of a biochemical reaction with cooperativity^4^; it expresses the occupancy *o*_*j*_, i.e. the fraction of enzyme bound to compound *j*, at a given compound concentration *c* (equation (1A)). Under the constraint that the binding of a compound *j* to a molecule of enzyme affects the enzymatic activity in a binary way, the occupancy *o*_*j*_ equals the fractional (enzymatic) effect *E*_*j*_(*c*), i.e. the observed effect relative to the maximum enzymatic effect.

1A $${o}_{j}={E}_{j}(c)=\frac{1}{1+{(\frac{{i}_{j}}{c})}^{{h}_{j}}}$$

where *i*_*j*_ and *h*_*j*_ are the inflection point (half maximal effective concentration, EC_50_) and the Hill or cooperativity coefficient, respectively. The equation was derived to describe a single-step enzymatic reaction with positive (*h*_*j*_ > 1) or negative (*h*_*j*_ < 1) cooperativity, but is often used to describe other, more complicated assays, for instance a cellular assay. In practice a four-parameter log-logistic function (4PLL), which amounts to a linearly transformed version of the occupancy *o*_*j*_, is used to directly fit the assay readout values *R*_*j*_(*c*) (equation (1B)). For a recent mechanistic derivation of this equation, see Mager 2003^5^.

1B $${R}_{j}(c)=b+({m}_{j}-b)\,{o}_{j}=b+\frac{{m}_{j}-b}{1+{(\frac{{i}_{j}}{c})}^{{h}_{j}}}$$

The above equation contains an intercept *b* that denotes the activity when no compound is bound to enzyme (at *c* = 0) and a scaling factor (*m*_*j*_ − *b*), where *m*_*j*_ is the maximal effect of the compound. Note that the baseline activity *b* is the same for all compounds, because it refers to *o* = *c* = 0, which reflects absence of any compound. Equations (1A) and (1B) describe occupancy and readout as a function of concentration of a given compound, respectively. When used in the context of compound combination studies, equations (1A) and (1B), which apply to single compounds, are called the marginal (or mono-therapy) concentration response functions.

### Inverse Hill equation: concentration as function of occupancy or readout

By simple rearrangement an inverse marginal function can be derived that describes concentration as a function of occupancy (equation (2A)) or readout (equation (2B)), respectively.

2A $${C}_{j}(o)={i}_{j}{({o}^{-1}-1)}^{\frac{-1}{{h}_{j}}}$$

2B $${C}_{j}(r)={i}_{j}{((\frac{{m}_{j}-b}{r-b})-1)}^{\frac{-1}{{h}_{j}}}$$

### Classical Loewe or Concentration Additivity model

The classical Loewe model (equation (3A)) describes the occupancy of enzyme exposed to a combination of two or more compounds. More specifically, occupancy is defined as the fraction of enzyme bound to any of the compounds in the mix. Under the first classical Loewe constraint that binding of compound to molecules of enzyme affects their activity in a binary (on/off) way, occupancy equals the fractional enzymatic effect of the mix of compounds.

3A $$\sum _{j}\frac{{c}_{j}}{{C}_{j}(o)}=1$$

In equation (3A), *c*_*j*_ is the concentration of compound *j* in the combination, and *C*_*j*_(*o*) is the concentration of compound *j* that would result in occupancy *o* when the compound would be administered as mono-therapy. Upon using equation (2A), equation (3A) becomes

3A1 $$\sum _{j}\frac{{c}_{j}}{{i}_{j}}{({o}^{-1}-1)}^{\frac{1}{{h}_{j}}}=1$$

By numerical solution of equation (3A.1), the occupancy can be computed under the assumption of Loewe Additivity^6^. Note that only the parameters of the Hill equation for the mono-therapies are required.

Analogously, equation (3B) describes the classical Loewe Additivity (LA) model as a function of readout.

3B $$\sum _{j}\frac{{c}_{j}}{{C}_{j}(r)}=1$$

Upon using equation (2B), equation (3B) becomes

3B1 $$\sum _{j}\frac{{c}_{j}}{{i}_{j}}{((\frac{{m}_{j}-b}{r-b})-1)}^{\frac{1}{{h}_{j}}}=1$$

which is the unnormalized form of the null model as derived by Greco^7^.

### Biochemically Intuitive Generalized Loewe (BIGL)

In practice, it sometimes occurs that the maximal responses of individual compounds in the same assay differ. Such cases cannot be reconciled with classical Loewe’s first constraint, which stipulates that the binding of a molecule of enzyme to any compound in the mixture results in an identical switch of binary activity state of that molecule. Indeed, this first constraint implies a single maximal response, reflecting saturation of all enzyme with compound. However, we propose a generalization of the Loewe model by allowing for varying and fractional compound effects at the enzyme molecule level. The classical Loewe model (equation (3A)) remains valid to describe enzyme occupancy, i.e. the fraction of enzyme bound to compound, because this equation does not refer to the (possibly compound-specific) maximal readouts *m*_*j*_. However, the linear transformation that maps occupancy to readout (equation (1B)) can be compound-specific: the scaling factors (*m*_*j*_ − *b*) then also account for variable and fractional compound effects. Yet, these scaling factors cannot be applied directly to the readout of combination activities. Our solution arises from the inherent properties of the classical Loewe model in that the readout of a combination is the sum of the readouts that are caused by the individual compounds in the combination. Hence, the readout of a combination can be written as a weighted average of the compound-specific readouts of equation (1B); this gives equation (3C)

3C $$r(o)=\sum _{j}\,{f}_{j}(o){r}_{j}$$

where weight *f*_*j*_(*o*) is the fraction of enzyme that are bound to compound *j* among those bound to any compound in the combination (with occupancy *o*). This fraction is exactly the interpretation that is given to the term $\frac{{c}_{j}}{{C}_{j}(o)}$ in the additive Loewe model (equation (3A)). Since by equation (3A) the sum of the ${f}_{j}(o)=\frac{{c}_{j}}{{C}_{j}(o)}$ equals 1, equation (3C) correctly expresses a weighted average. Upon using equations (1B) and (2A), equation (3C) becomes

3D $$r(o)=b+o\sum _{j}({m}_{j}-b)\frac{{c}_{j}}{{i}_{j}}{({o}^{-1}-1)}^{\frac{1}{{h}_{j}}}$$

In summary, the BIGL model is applicable for combinations of compounds with different maximal responses. It is described by equations (3A) and (3D). The former expresses Concentration Additivity (classical Loewe) and is used for the calculation of the occupancy. The latter is subsequently used for the prediction of the readout *r* for the combination experiment under Loewe. Note that the BIGL model only requires parameter estimates from the marginal concentration response curves to predict the null readout of compound combinations.

### Two statistical tests for synergy

In a synergy experiment compounds are tested in several concentration combinations. If the assay readout values deviate too strongly from what is predicted under BIGL model (here referred to as the null model), the combination effect is called synergistic or antagonistic. Statistical tests are designed to measure the evidence in the data against the null model in the presence of variability. Two hypothesis tests are proposed here. Both tests basically contrast the observed readouts of the combination experiments with those predicted from the null model (equations (3A) and (3D)). The only unknown parameters in the null model originate from the Hill equations (equation (1B)), and these are estimated from the mono-therapy data. The first test assesses the average deviation from the null model, and is referred to as the MeanR test. The second test makes use of the largest absolute deviation between observed and predicted readout among all concentration combinations, and allows for the identification of concentration combinations at which synergy or antagonism is present. This test is referred to as the MaxR test. Details about the construction, assumptions and theory of the statistical tests are presented in Supplementary Methods. Results from a simulation study demonstrate the validity of the two testing procedures; see Supplementary Results (Supplementary Figures S1 and S2).

### Evaluation of the method

**Figure 1:**
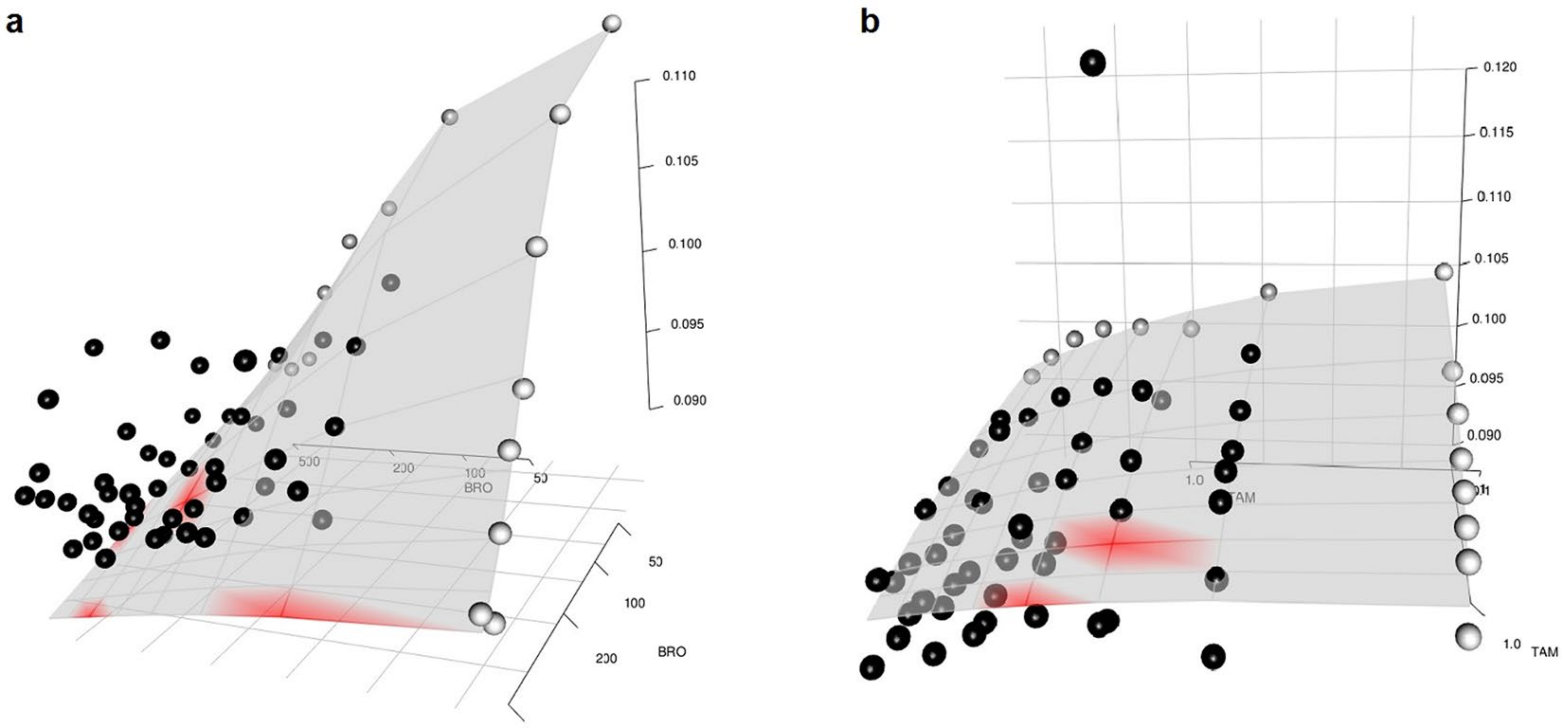
Response surface plots with an indication of synergy/antagonism by blue or red respectively. (**a**) Sham combination of BRO at 8 hours with indication of some areas of antagonism at high dose ranges. (**b**) Sham combination of TAM at 8 hours with indication of antagonism but driven by one extreme outlier.

First, we evaluated the BIGL methodology on some sham (i.e. self) combinations, which by definition should be additive. We used the sham combinations of 25 compounds as presented in Cokol *et al*.^8^. After filtering, based on the variance on the estimated log(EC50) to remove poor quality data (see Methods), 14 compounds were left and their sham combinations were analyzed both at 8 hours as well as 12 hours. Of the 28 sham combinations tested, 4 (14%) were called non-additive, which is slightly higher than expected at the 5% significance level (Table 1). The null was rejected for all 4 combinations in favor of an antagonism call. However, the combination data of both BRO (8 h) and MET (8 h, 12 h) are showing lower effects than expected under the null at higher dose ranges (Fig. 1a). This implies either deviation from additivity or data quality issues (e.g. due to toxicity). The TAM combination is also called antagonistic, but this call is clearly driven by one extreme outlier (Fig. 1b). In summary, after accounting for outliers and data quality issues, the BIGL call rate for deviation of additivity on sham combinations is in line with expectations.

**Table 1:** Tested sham combinations of Cokol *et al*.^8^ at 8 hours and 12 hours.

Time	ANI	BRO	C3P	CAN	CYC	HAL	MET	MMS	MYR	QMY	RAP	STA	TAM	TUN
08h00	0	1	0	0	0	0	1	0	0*	0	0	0	1	0
12h00	0	0	0	0	0	0	1	0	0	0	0	0	0	0

0 indicates additive call, 1 indicates antagonism and -1 indicates synergy. 4 sham combinations are found to be antagonistic. *For Myr-Myr combination the p value of MeanR was borderline significant (p = 0.047), but no significant calls for MaxR.

Next, we evaluated the performance of the BIGL methodology on a larger combination dataset, namely the OncoPolyPharmacology Screen published by O’Neil^9^. We successfully executed BIGL analyses on all combination data provided by O’Neil (Supplementary Figures S5 and S6 present an overview by heatmap). As a quality control prior to further analysis, we applied a similar filtering as used in the Cokol dataset (see Methods) which led us to invalidate 72% of the initial combinations (583 pairs of compounds tested in a varying subset of 39 cell lines). 438 (75%) compound pairs were found synergistic in at least one cell line under the BIGL model, which is more than the 50% in the O’Neil study, which reports analyses for the Highest Single Agent (HSA) and Bliss null models.

**Figure 2:**
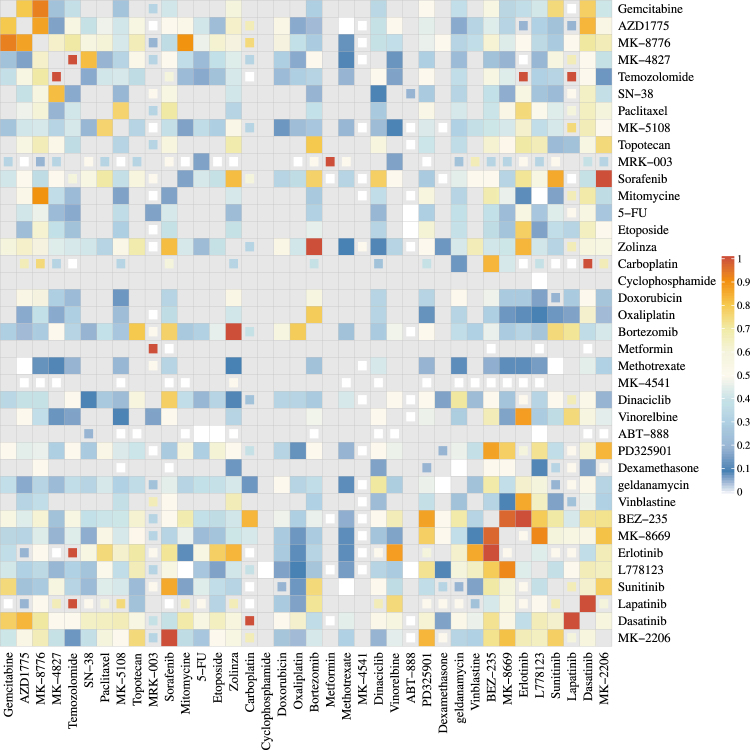
Heatmap illustrating synergistic combinations. The color indicates the fraction of cell lines for which a synergy call was made, accounting for the respective number of cell lines analyzed for a particular pair. Fractions based on fewer than 6 cell lines are minimized. Grey heatmap cells represent compounds pairs absent from the dataset.

Figure 2 visualizes the fraction of cell lines with synergy calls for each compound pair. For convenience, the ordering of the compounds is kept consistent with the heatmap in the O’Neil study. 181 combinations (31%) were called synergistic in half of the analyzed cell lines. The O’Neil study highlighted the combination of Wee1 inhibitor (AZD1775) with MK-8776 as a consistently strong synergistic drug combination, a finding confirmed in Guertin *et al.* ^10^. The combination data for this compound pair in 15 out of 39 cell lines passed data quality criteria; the BIGL evaluation returned a synergy call for 13 of those, in line with the O’Neil conclusions. The O’Neil study also reports synergy of the pairing of the same Wee1 inhibitor (AZD1775) with an mTOR inhibitor (MK-8669) in roughly 30% of the cell lines. The combination data for this compound pair in 18 out of 39 cell lines passed data quality criteria, and the BIGL approach called synergy for 6 (33%) of those.

**Figure 3:**
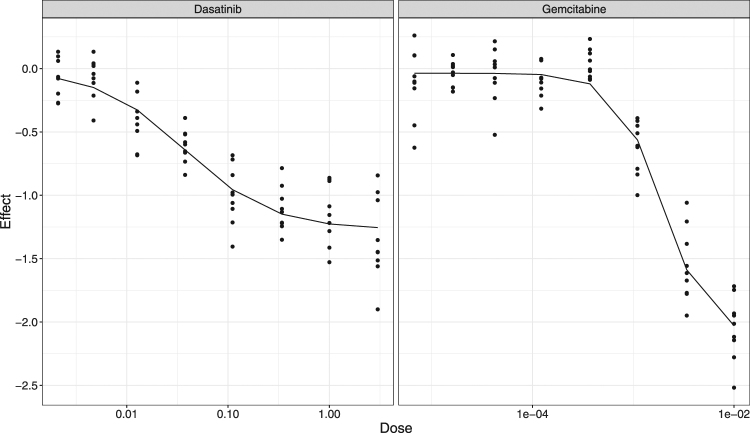
Mono-therapy plots. Mono-therapy data in LOVO cell line for Gemcitabine and Dasatinib.

**Figure 4:**
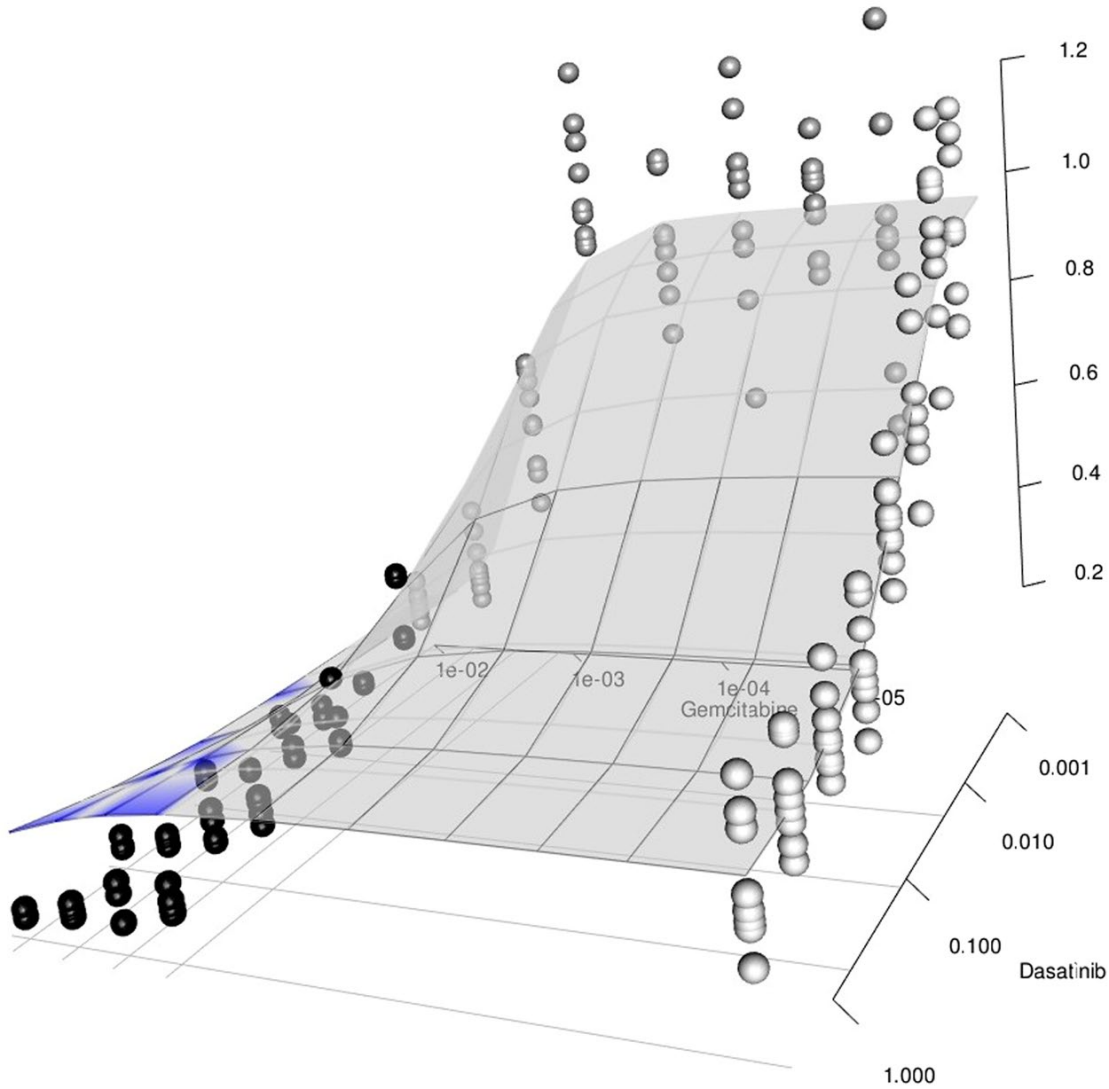
Response surface of gemcitabine and dasatinib in LOVO cell line. At high dose range of the two compounds, the combination data show effects below the expected ones under the null, indicating synergistic effects.

Additionally, the BIGL approach also flagged some combinations that were reported as synergistic elsewhere but that were not picked up in the O’Neil study. Figure 3 (marginal dose-response curves) and Fig. 4 (combination dose-response surface) illustrate the analysis of one such case, namely the combination of Gemcitabine and Dasatinib in the LOVO cell line. These figures also visualize the hallmark ability of the BIGL methodology to accommodate the partial agonism of Dasatinib (Fig. 3), which classical Loewe handles poorly. In line with findings elsewhere^11^, BIGL flags the combination of Gemcitabine and Dasatinib as synergistic in 14 out of the 18 cell lines with quality controlled datasets.

## Discussion

With the advent of combination therapy (for a recent review, see e.g. He *et al.* ^12^), the treatment options for many diseases have drastically improved. The Loewe or Concentration Additivity is often used as a biochemically interpretable null model for an early assessment of the potential of combinations. Even though the very simple biochemical reaction mechanism that underlies Loewe is in most cases (e.g. in a cellular or organism context) incomplete at best, it still enables the straightforward computation of an easy-to-interpret baseline expectation. This computation only requires routinely available concentration-response curves of the individual compounds, and no mechanistic information that is typically unavailable during early drug discovery. Used in a synergy call context, the Loewe null model enables the prioritization of compound combination that deviate favorably from the expectation. These per definition reject Loewe as an accurate mechanistic model. For the most interesting combinations, a mechanistic model can be elaborated that explains the deviation from the Loewe null, but this typically requires considerably more data generation.

The classical Loewe reference model is not applicable whenever the concentration-response curves of the compounds in the combination have different maximal effects, like in pairings of an agonist with a partial or an inverse agonist. Whereas several methods that build on classical Loewe have been developed to address these issues (Table 2), to the best of our knowledge, BIGL is the first method to handle combinations with partial and inverse agonists (see Supplementary Figure S3 and Flaveny *et al.* ^13^) that preserves the biochemical interpretability of the original null model. In this paper, we have derived the model equations of the BIGL method and developed statistical tests for synergy testing. Moreover, we demonstrated the amenability of the BIGL approach to a real world large oncology screen of combinations which contains clear examples of combinations with partial agonists^9^. The BIGL obtained synergy assessment results on this screen were generally consistent with literature. Additionally, we showed through simulation (Supplementary Results) and performance on self combinations that the type I error rate is controlled in general.

**Table 2:** Classical LA and models that generalize classical LA for partial agonism.

Method	Year published	biochemically interpretable	Comments	Tool
**A. Classical Loewe (Concentration Additivity)**				
Loewe^3^	1926	✓	not applicable for marginal curves with different maximal effect	R package *BIGL*
**B. LOF-test of null models**				
GCA (Howard)^19^	2009		assumes hill slope = 1 for the marginal curves	
Scholze^20^	2014		rescales curves to common effect levels	
FLM^23^	2016		special cases are compatible with Loewe	
Loewe (Di Veroli)^21^	2016		reverts to highest single agent where classical Loewe is undefined	MATLAB *Combenefit*
BIGL	2017	✓		R package *BIGL*
**C. Response surface capture models**				
Harbron^22^	2010		explores multiple null models	
BRAID^24^	2016		BRAID additivity surface is not Loewe additive	R packages *braidrm* and *braidReports*

Like classical Loewe, BIGL posits that compounds compete for the same enzymatic binding site (otherwise its results would deviate from classical Loewe where this applies). The combination response reflects the sum of the difference between maximal and baseline response for each compound, weighted by the fraction of (virtual) enzyme bound to that compound. Hence, the most extreme maximal response of any single compound in a combination also defines the most extreme maximal response of that combination, which is achieved by saturation of the enzyme with that compound^14,15^. The response of combinations that include the same concentration of a full agonist (i.e. the compound with the most extreme maximal response) will be reduced with increasing concentrations of a partial agonist (i.e. the compound with a less extreme maximal response). Both in clinical applications and in discovery, an agonist compound can be combined with a neutral antagonist (which competitively blocks the agonist without being enzymatically active) or an inverse agonist (which triggers an enzymatic response in the other direction, for instance in assays with a constitutively active receptor, channel or transporter, an inverse agonist would reduce such activity). Known clinical compound combinations target the μ opioid receptor and the α1 adrenergic receptor^16,17^. To illustrate the flexibility of BIGL, we simulated the expected response of a combination of a partial opioid receptor agonist with a neutral antagonist with dose response taken from^18^ (Supplementary Figure S4). The case of a neutral antagonist presents the extreme case where one of the combination compounds show a maximal response equal to baseline. Notably, if a compound combination is specified as a combination of *individually active* compounds, then it could be argued to opt for the alternative null model of the single active compound. A straightforward test of a null hypothesis of non-activity for both compounds would enable to automate the identification of such cases.

As stated above, BIGL is not the first method that generalizes the classical Loewe model. The currently existing methods that generalize the classical Loewe model to deal with partial and/or inverse agonisms are listed in Table 2. Some of these methods overextend conventional methods beyond their justified application domain by simply ignoring inconvenient response curve or compound combination characteristics, even if these are very real. Thus, the Generalized Concentration Addition (GCA) method of Howard^19^ imposes Hill coefficients of 1 for all compounds and thereby ignores possible cooperativity. The extrapolation method described in Scholze^20^ deals with partial agonism by rescaling any maximal effects to the overall most prominent maximal effect, hereby ignoring observable differences in maximal effect. A piecewise approach is described in^21,22^, where contributions of compounds are accounted for only within their individual effect range, but completely ignored outside of it. As a general guideline, it is advised to deploy methods that correctly consider observed data characteristics.

Another group of methods was designed to address different maximal responses properly. The FLM model^23^, for example, is similar to BIGL in that its function is constructed as a weighted average of terms that are scaled to the original marginal dose-response functions. However, it conceptually hinges on effect equivalence concepts that implicitly assume parallel concentration-response curves (with identical Hill coefficients and maximal responses). Under those constraints and only then, the FLM model is a special case of the LA model, and by extension the BIGL model. In all other cases, the model is mathematically convenient, but biochemically uninterpretable. The BRAID model of Twarog *et al.* ^24^ is another method that adheres to the LA constraints, thus building on the Hill dose response for the individual compounds and satisfying the Loewe self-combination additivity property. However, the BRAID additivity surface is not Loewe additive^24^. Therefore, unlike BIGL, it never reduces to LA conceptually which limits interpretability. Moreover, as the BRAID model presents a mathematical solution to deal with partial agonists, the modeled concentration addition can involve negative concentrations which is biochemically unintuitive.

Besides different ways of generalizing the classical Loewe model, the methods listed in Table 2 also differ in the way they assess synergy. They can be divided in two types: response surface modeling and lack of fit testing of the null model. Response surface models capture the deviation of the observed response surface from the null model relying on the parameters of the marginal dose response functions and one additional parameter that enables synergy antagonism calling. For example, the BRAID model^24^ comprises a single interaction parameter *κ*, which is statistically equivalent to 0 under additivity, but deviates under synergy or antagonism. In addition to enabling compound interaction calls, well fitted response surface models can be used for interpolating the effect of unobserved compound combinations. However, while a single interpretation parameter supports straightforward compound interaction calling, the resulting models often lack the flexibility to accurately adjust the observed response surface. For instance, they impose that over the entire concentration range the effects of the compounds in the mixture occur in the same direction – either consistently positive or consistently negative^6^. A better fit and hence interpolation performance can be achieved by exploring a bigger modeling space. Thus, multiple reference models can be evaluated^22^, and the compound interaction call based on the best fitting one. However, this may encumber the comparison of combinations, if optimal fitting selects different models as reference for the compound interaction assessment. In practice, response surface model methods often face a trade-off of interpolation performance versus consistency of interaction assessment. Notably, optimal predictivity typically benefits from more extensive model flexibility, as illustrated by mechanism based models (outlined in^5^). However, these models are not designed to support a comparison to a null model, which is different from the second group of methods: lack of fit models where compound interaction evaluation only aims to assess the deviation from the null model, informally or formally (i.e. statistically). Early examples include various isobologram approaches, which evaluate deviation of the null model for isoeffective sections through the dose response surface. Isobologram analyses have been extended to the partial agonist case^25^.

The proposed statistical tests within the BIGL framework are lack of fit methods. They rely on a null model that, like the Loewe model, requires no other parameterization than the parameters of the concentration-response curves of the individual compounds and as assumptions the ones underpinning the standard practice of fitting a four-parameter log-logistic dose-response function or Hill equation (which generalizes the Michaelis-Menten equation to accommodate cooperativity as Hill coefficients deviating from one). Additionally, the BIGL lack of fit tests assume equality of variance of outcomes – however this could be relaxed in future developments. BIGL naturally generalizes Loewe by allowing for different maximal responses. Even though the Loewe and consequently BIGL models are inspired by a specific and simple enzymatic mechanism, when used in the context of synergy or antagonism call, they are not assumed to be mechanistically accurate. Like Loewe, BIGL is straightforward to compute with minimal and routinely available information (concentration-response curves), and enables to select combinations of interest. Importantly, the most attractive compound combinations would typically be the ones that deviate favorable from the BIGL expectation, and thereby reject BIGL as the mechanism of action.

In summary, we propose BIGL as a tractable and flexible approach to assess compound interaction with minimal and routinely available data requirements, that naturally generalizes the widely used Loewe model and retains Loewe’s biochemical interpretability.

## Methods

### Datasets

The BIGL methodology was applied on two different publicly available datasets. The first dataset, which was described in Cokol *et al*.^8^, contains 25 sham (self) combinations provided for each compound combination as raw cell growth measurements in a full, 8 concentration checkerboard design (8 × 8) in 24 hour time course with 15 minute intervals. In the current study data for the 8 and 12 hour timepoints were analysed.

The second dataset came from the OncoPolyPharmacology screen as described in O’Neil *et al*.^9^, which describes the assessment of 38 drugs (22 experimental and 16 approved drugs) in a panel of 39 cancer cell lines for a total of 583 pairs. The mono-therapy data are expressed as cell growth rate relative to the growth rate of DMSO-treated controls.

### Estimation details

For the mono-therapy data for each compound pair, a 4-parameter log-logistic function was fitted, subject to the constraint that both compounds share the same baseline response. Additionally, to stabilize residual variance during the marginal parameter estimation procedure, the readouts were log-transformed to instantaneous growth reads^26^. Parameters were estimated by a non-linear least squares approach. A model was considered of sufficient quality if the standard deviation of log-transformed half-maximally effective concentration (log(EC50)) estimates for both compounds in a pair did not exceed 10.

The marginal parameters were subsequently used to construct the response surface as predicted by the generalized Loewe model and MeanR and MaxR statistics were computed from the predicted and observed off-axis data. Null distributions for these statistics were obtained assuming normally distributed off-axis residuals for all compound pairs in the O’Neil *et al*. dataset (see Supplementary Methods). In contrast, a bootstrapping method with 1000 runs was used for the Cokol *et al*.^8^ dataset to approximate null distributions of the MeanR and MaxR statistics. Since no replicates were available in the latter dataset, the readout errors for off-axis points during bootstrapping were sampled from a centered Gaussian distribution with variance equal to the residual variance estimate from the marginal model. In all of the above cases, the *C*_*p*_ matrix (see Supplementary Methods) was calculated using 100 bootstrap runs and all statistical tests were performed at a significance level of 5%.

## Electronic supplementary material

Supplementary Material
