# Supplementary material for: BIGL: Biochemically Intuitive Generalized Loewe null model for prediction of the expected combined effect compatible with partial agonism and antagonism
Source: Sci Rep. 2017 Dec 20;7:17935. doi: 10.1038/s41598-017-18068-5 (PMC5738392; doi:10.1038/s41598-017-18068-5)
Supplement: Supplementary file 1 — Supplementary Material [file 41598_2017_18068_MOESM1_ESM.pdf]

Supplementary Material:

# BIGL: Biochemically Intuitive Generalized Loewe null model for prediction of the expected combined effect compatible with partial agonism and antagonism.

Koen VAN DER BORGHT[1], Annelies TOURNY[2], Rytis BAGDZIUNAS[3], Olivier THAS[2,4], Maxim NAZAROV[3], Heather TURNER[3], Bie VERBIST[1] & Hugo CEULEMANS[1]

[1] Discovery Sciences, Janssen Research & Development, Turnhoutseweg 30, B-2340 Beerse, Belgium

[2] Department of Mathematical Modeling, Statistics and Bioinformatics, Ghent University, Coupure Links 653, B-900 Gent, Belgium

[3] Open Analytics, Jupiterstraat 20, B-2600 Antwerpen, Belgium

[4] National Institute for Applied Statistics Research Australia (NIASRA), University of Wollongong, Northfields Avenue, Wollongong NSW 2522, Australia

# Supplementary information BIGL paper

## 1 Supplementary methods

Two statistical tests were designed to measure whether a combination of different compounds results in a synergistic or antagonistic effect. The first test, referred to as MeanR, tests the overall fit of the data to the Generalized Loewe additivity model (further referred to as the null model). If the assay readout values deviate too strongly from this null model, then one may identify synergy or antagonism. The size of the deviation that is allowed under the null model depends on the biological variability present in the data.

A second test, MaxR, allows the identification of combinations of concentrations where synergy or antagonism is present by comparing the absolute deviation between observed and predicted readout. Both tests are tests of the null hypothesis that the null model holds true.

The following sections describe the derivation of the test statistics MeanR and MaxR, as well as their null distributions required for p-value calculations.

### 1.1 Derivation of the MeanR test statistic

The null hypothesis states that the Generalized Loewe additivity model holds true. The parameters in this model come from the Hill equations for the mono-therapies. Hence, only the observations from the mono-therapies are needed to fit the model. The compound concentrations for the mono-therapies are referred to as the $n_0$ on-axis points. The residual variance is denoted by $\sigma_0^2$. Based on the fit an estimate of the residual variance is computed as the mean squared error, which is denoted by $MSE_0$. The fitted model is further used to predict the $n_1$ assay readouts at the concentrations of the compound combinations, which are referred to as off-axis points.

We use the index $i$ to refer to an off-axis point. We first describe the method for the setting where no replicate measurements are available at the $n_1$ off-axis points. Let $R_i$ denote readout at off-axis point $i$, and let $\hat{R}_i$ denote the predicted readout at off-axis point $i$ according to the null model. We assume that all readouts are independently distributed. Under the null hypothesis the off-axis observations $R_i$ come from the same null model, with possibly a different residual variance, say $\sigma_1^2$. Our test is based on the residuals

$$E_i = R_i - \hat{R}_i.$$

In matrix notation we write

$$E = R - \hat{R}$$

(i.e. vectors constructed by stacking $E_{ij}$, $R_{ij}$ and $\hat{R}_i$). The covariance matrix of $E$ is then given by

$$\text{Var}(E) = \text{Var}(R) + \text{Var}(\hat{R}) = \sigma_1^2 I + \sigma_0^2 C_p,$$

where $C_p$ is the covariance matrix of the predictions.

For a linear model, $C_p$ can be easily computed from the design matrices of the data used to fit the null model ($X_0$) and of the off-axis points of the predictions ($X_1$):

$$C_p = X_1(X_0^t X_0)^{-1} X_1^t,$$

but for a nonlinear model $C_p$ has no simple expression. We propose to approximate $C_p$ by means of a bootstrap procedure (see further).

Under the assumption of normality, so far we have

$$E \sim MVN(0, \sigma_1^2 I + \sigma_0^2 C_p).$$

Hence, a meaningful test statistic is the quadratic form

$$E^t(\sigma_1^2 I + \sigma_0^2 C_p)^{-1} E.$$

However, the residual variances $\sigma_0^2$ and $\sigma_1^2$ are unknown. With $MSE_0$ and $MSE_1$ their corresponding consistent estimators, the test statistic

$$E^t(\mathrm{MSE}_1 I + \mathrm{MSE}_0 C_p)^{-1} E$$

is asymptotically distributed as $\chi^2_{n1}$ under the null hypothesis, but the convergence may be slow.

An exact null distribution may be obtained by making the additional assumption that $\sigma_0^2 = \sigma_1^2$. This common variance is denoted by $\sigma^2$. We then get

$$E \sim MVN(0, \sigma^2(I + C_p)),$$

and the quadratic form becomes

$$E^t\left(\sigma^2(I + C_p)\right)^{-1} E = \frac{E^t(I + C_p)^{-1}E}{\sigma^2} \sim \chi^2_{n1}.$$

Suppose we have an estimator of $\sigma^2$, say $S^2$ (details follow later), then it is often possible to show (for a linear model, and under the assumption of normality of the model error terms)

$$m\frac{S^2}{\sigma^2} \sim \chi^2_m,$$

for an appropriate $m$. The test statistic may now be written as

$$\frac{E^t(I + C_p)^{-1}E}{S^2} = n_1 \frac{E^t\left(\sigma^2(I + C_p)\right)^{-1} E/n_1}{(mS^2/\sigma^2)/m},$$

in which we recognize in the numerator a statistic with distribution $\chi^2_{n1}/n_1$, and in the denominator a statistic with distribution $\chi^2_m/m$. If numerator and denominator are independent, then by definition this ratio is distributed as $F_{n1,m}$. Hence we write

$$T_{MeanR} = \frac{E^t(I + C_p)^{-1}E}{n_1 S^2} = \frac{E^t\left(\sigma^2(I + C_p)\right)^{-1} E/n_1}{(mS^2/\sigma^2)/m} \sim F_{n1,m}.$$

Thus, if we find an estimator $S^2$ of $\sigma^2$ for which (A) $mS^2/\sigma^2 \sim \chi^2_m$ and (B) which is independent of $E$, we can use $T_{MeanR}$ as a test statistic with null distribution $F_{n1,m}$. In this paper we propose to use $S^2 = MSE_0$ as the estimator; under the normality assumption (A) holds with $m = df_0$ (residual degrees of freedom from the fit of the null model using only the on-axis points), and since $MSE_0$ only makes use of

the mono therapy data, it is definitely independent of the residuals $E$ that are calculated from the off-axis data. Thus the final form of the test statistic is given by

$$T_{MeanR} = \frac{E^t(I + C_p)^{-1}E}{n_1 \mathrm{MSE}_0} = \frac{E^t\left(\sigma^2(I + C_p)\right)^{-1}E/n_1}{(df_0 \mathrm{MSE}_0/\sigma^2)/df_0} \sim F_{n1,df0}.$$

Thus, if the residuals are normally distributed and if the residual variances at the off-axis and on-axis (mono-therapies) points are equal, it can be shown that $T_{MeanR}$has approximately an $F_{n1,df_0}$ null distribution. Where $df_0$ equals the degrees of freedom of $\mathrm{MSE}_0$.

If no normality can be assumed, then the null distribution can be obtained through the parametric bootstrap (see further).

### 1.1.1 MeanR in the presence of replicates

The previous definition holds when no replicates are included in the study. When replicates are available at the off-axis points, we continue to work with the average residuals at the off-axis points. In particular, let $\overline{R}_i$ denote the average readout of the $m_i$ replicates at off-axis point $i$. We then define the (average) residual at off-axis point $i$ as

$$E_i = \overline{R}_i - \hat{R}_i.$$

Let $E$ denote the vector with all the $E_i$ stacked. Then, under the null hypothesis of no lack-of-fit and assuming constant variance,

$$E \sim MVN(0, \sigma^2(D + C_p)),$$

with $D$ a diagonal matrix with at the $i$th position $1/m_i$.

The test statistic and its null distribution can now be obtained in a similar way as before. In particular,

$$T_{MeanR} = \frac{E^t(D + C_p)^{-1}E}{n_1 \mathrm{MSE}_0} = \frac{E^t\left(\sigma^2(D + C_p)\right)^{-1}E/n_1}{(df_0 \mathrm{MSE}_0/\sigma^2)/df_0} \sim F_{n1,df0}.$$

If no replicates are available, the $D$ matrix becomes the identity matrix $I$ and the method reduces to the one described in the previous section.

The $C_p$ matrix will be estimated using the bootstrap method, and to avoid relying on the normality assumption a parametric bootstrap method will be used for p-value calculation.

### 1.1.2 Bootstrap method to estimate the $C_p$ matrix

Since the BIGL null model is highly non-linear, we propose a parametric bootstrap procedure to approximate the $C_p$ matrix:

1. pool all residuals of the on-axis and off-axis points
2. for each on-axis point *i*, sample (with replacement) $m_i$ residuals completely at random from the pooled sample (step 1)
3. add residuals to corresponding predicted readout from fitted Hill equations (mono-therapies); this generates a bootstrap sample of readouts at the on-axis points

4. refit the Hill equations to the bootstrap sample data
5. use the fit from step 4 to generate predictions at the off-axis points
6. repeat steps 2-5 many times (say at least 100 times) and compute the covariance matrix between the off-axis predictions. This gives an approximation of the $C_p$ matrix.

### 1.1.3 Bootstrap method to estimate the null distribution of $T_{MeanR}$

If no normality can be assumed, then the null distribution of the $T_{MeanR}$ test statistic can be obtained through a parametric bootstrap procedure. This procedure is very similar to the bootstrap procedure outlined in the previous section. In particular, steps 1-5 are identical, but the remaining steps are now:

6. Sample completely at random $m_i$ residuals from the pooled sample of residuals (step 1) for each off-axis point *i*. Add these sampled residuals to the predicted readout. This forms a bootstrap sample of readouts at the off-axis points (under the null hypothesis of additivity)

7. Compute the test statistic based on the bootstrap sample of step 6.

8. Repeat steps 2-7 many times. The set of test statistics computed in step 7 forms the bootstrap null distribution of the test statistic and can be used for p-value calculations.

Note that in the calculation of the test statistic (step 7) the matrix $C_p$ is required. Hence, the final procedure is a nested bootstrap method in which the bootstrap procedure of section 1.1.2 is included in step 7. However, from experience (simulation studies) we have learnt that the matrix $C_p$ is quite stable. Therefore, as an approximation, we suggest to compute $C_p$ only once and use this single $C_p$ approximation throughout the bootstrap procedure as outlined in this section.

## 1.2 Derivation of the MaxR test statistic

We start from the residuals defined for MeanR:

$$E_i = \overline{R}_i - \hat{R}_i.$$

Let $E$ denote the vector with all the $E_i$ stacked. Then, assuming normality, equal residual variance at off and on-axis points, and under the null hypothesis of no lack-of-fit,

$$E \sim MVN(0, \sigma^2(D + C_p)),$$

with $D$ a diagonal matrix with at the $i$th position $1/m_i$.

Define

$$T_{MaxR} = \max \mid E^t (D + C_p)^{-1/2} \mid / \sigma.$$

Then, under the null hypothesis,

$$T_{MaxR} \sim \max \mid Z_1, \ldots, Z_{n_1} \mid,$$

where $Z_1, \ldots, Z_{n_1}$ i. i. d $N(0,1)$.

Now we replace $\sigma$ in the definition of $T_{MaxR}$ with $\sqrt{\mathrm{MSE}_0}$,

$$T_{MaxR} = \max \mid E^t(D + C_p)^{-1/2} \mid / \sqrt{\mathrm{MSE}_0}.$$

Then, assuming normality, equal residual variance and under the null hypothesis,

$$T_{MaxR} \sim \max \mid Z_1, \ldots, Z_{n_1} \mid,$$

where $Z_1, \ldots, Z_{n_1}$ i. i. d $t_{df_0}$.

If the null hypothesis is rejected at the alpha-level of significance with the $T_{MaxR}$ test statistic, one can identify the off-axis points that were responsible for the rejection (i.e. the off-axis points at which the corresponding element in $E^t(D + C_p)^{-1/2}/\sqrt{(\mathrm{MSE}_0)}$ exceeds the critical value of the test). These points correspond to the compounds for which the readout is different from what is expected under additivity. This procedure basically performs simultaneous hypothesis tests at the individual off-axis points. The procedure controls the familywise error rate (FWER) at the nominal significance level of the $T_{MaxR}$ test.

## 2 Supplementary results

Monte-Carlo simulations were conducted to assess whether the MeanR method is able to control the type I error at a nominal level of 5%. Furthermore, we have assessed whether the MeanR method could truly detect synergy and whether the MaxR method is able to select the true synergistic off-axis points.

For each scenario, 3000 Monte-Carlo simulations were performed. All tests were conducted at the nominal 5% significance level. All p-values were computed with the parametric bootstrap (100 bootstrap runs).

### 2.1 Simulations under additivity

In each Monte-Carlo run, data were generated with parameters estimated from a real dataset, using the Generalized Loewe additivity model. Data of multiple replicates of on- and off-axis points were available in the dataset. The $C_p$ matrix is computed based on 50 bootstrap runs.

The results are presented in Figure S1. From the results we conclude that the test statistic follows indeed approximately an F-distribution and that the null distribution of the p-values is approximately uniform. The type I error rate is 6% which is close to the nominal level of 5%.

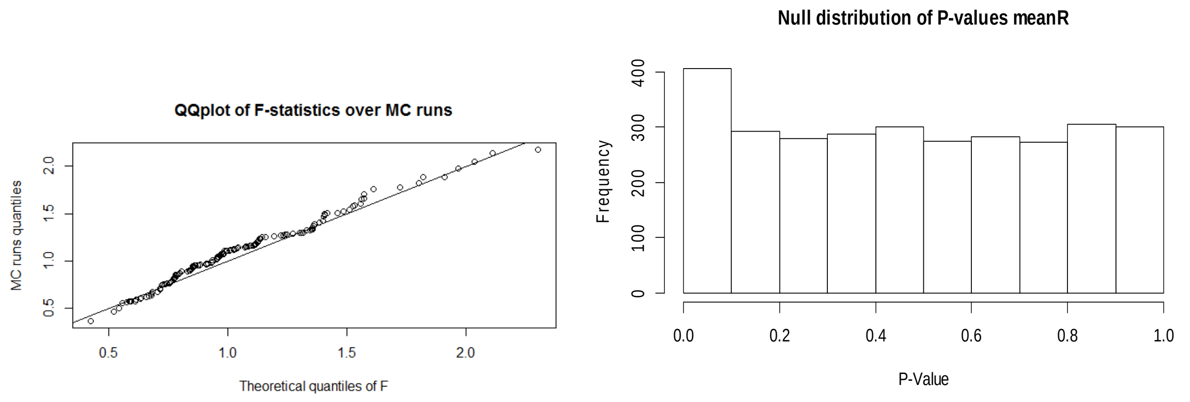


*Figure S1. On the left side a QQplot of the F-distribution of the test statistic is shown under additivity. On the right the null distribution of the p-values are shown*

## 2.2 Simulations under synergy

To verify whether the method is indeed able to detect synergy, we have simulated data with a synergistic effect at three dose combinations. Data were first simulated under the null hypothesis using the estimated parameters obtained from the Loewe model. Afterwards, a small effect of 0.075 was subtracted from the predicted effect under additivity. This process was repeated 3000 times and the bootstrap p-values of each test were saved.

Figure S2 shows that the MeanR method is indeed able to detect synergy at the $\alpha$-level of 5%. For the chosen effect of 0.075 we found that a synergy call was made in 63.9% of the Monte-Carlo runs (i.e. power of test equals 63.9% in this scenario). The power of the test increases with increasing effect size.

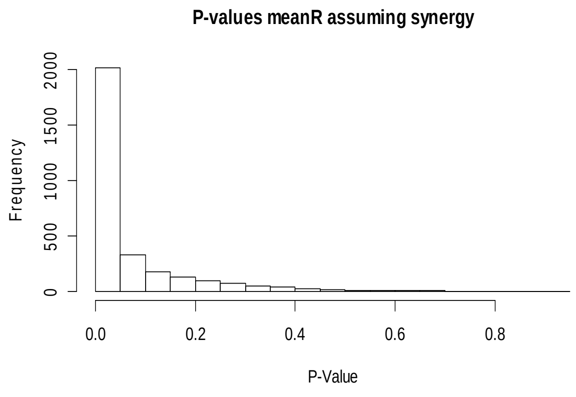


*Figure S2. Histogram of the p-values in the presence of synergy.*

## 2.3 Sensitivity and Specificity of MaxR

The test MaxR can detect those off-axis points with readouts different from what is expected under the additive model. To assess its performance, we have looked at the sensitivity and specificity of the test. The MaxR test was carried out only when the p-value of the MeanR test was < 0.05. Since we know exactly in which points we have inserted a synergistic effect, we can check whether the MaxR test is able to select these three points. The test was able to detect all true synergistic points in 52.06% of the cases (= sensitivity). The specificity of the test however was, 99.46%. We can conclude that for the chosen effect size of 0.075 the MaxR test almost never selects a negative point as a synergistic effect, but the method is only able to detect all synergistic points in 52.06% of the cases. If another balance between sensitivity and specificity is desired, ROC curves can be constructed by varying the p-value threshold used to call synergy/antagonism.

Another simulation study where the effect size was doubled (0.15) showed that the power of the MeanR test increased to 99%. The specificity of the MaxR test decreased a little to 99.21%. The sensitivity of the test however increased to 99.23%. This shows that the sensitivity and specificity of the test are strongly dependent on the effect size.

## 3 Shiny application

- An interactive shiny application (https://bigl.openanalytics.eu) visualizes the predicted response surface for a given set of marginal model parameters. Data points are simulated according to the BIGL null model from the provided marginal parameters. Marginal parameters are then re-estimated from the data and the response surface is subsequently computed and depicted as a 3-dimensional plot. Detailed predicted response decomposition in the BIGL model is available within the application as well. This interactive web application comes with 3 pre-defined examples of compound pairs, namely two agonists, an agonist and a partial agonist as well as an agonist and an antagonist (Figure S3).

Additionally one can use an alternative null model, being either the classical Loewe or the highest single agent (HSA) model. In the classical Loewe, both maximal responses of the marginals are restricted to be the same and the surface is computed. Expected response for the HSA, on the other hand, is constructed simply by taking either the minimum (if dose response curves are decreasing) or the maximum (if dose response curves are increasing) at a particular dose combination.

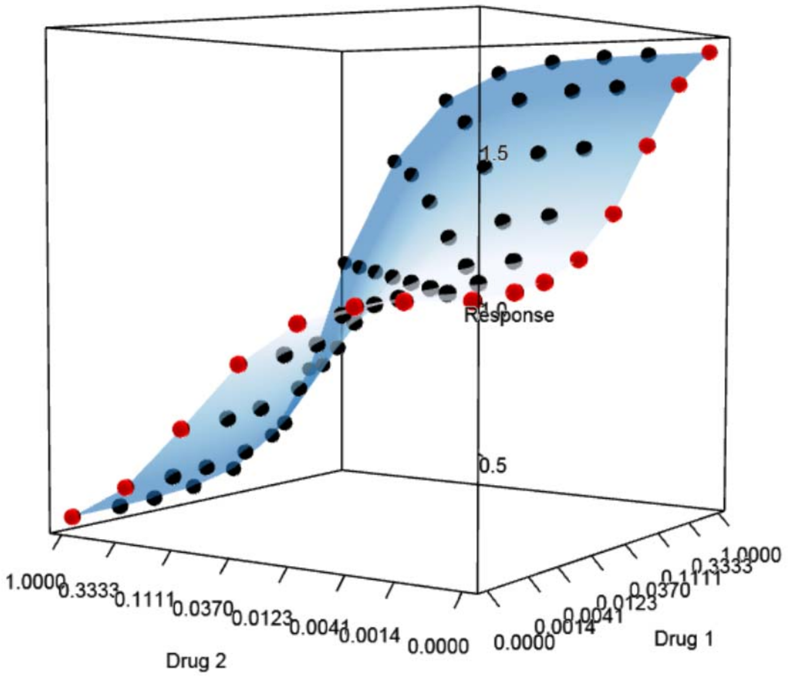


*Figure S3. Example BIGL null surface for Agonist-Inverse Agonist combination as obtained by R Shiny app.*

For additional interpretation of the BIGL null response surface, in the R shiny web application a corresponding two-dimensional visualization (isobologram using a colour scale) is made. As an example, in Figure S4 a dose response combination of a partial agonist and a neutral antagonist has been simulated using the BIGL model from the marginal dose response curves as shown below (doses are in linear scale).

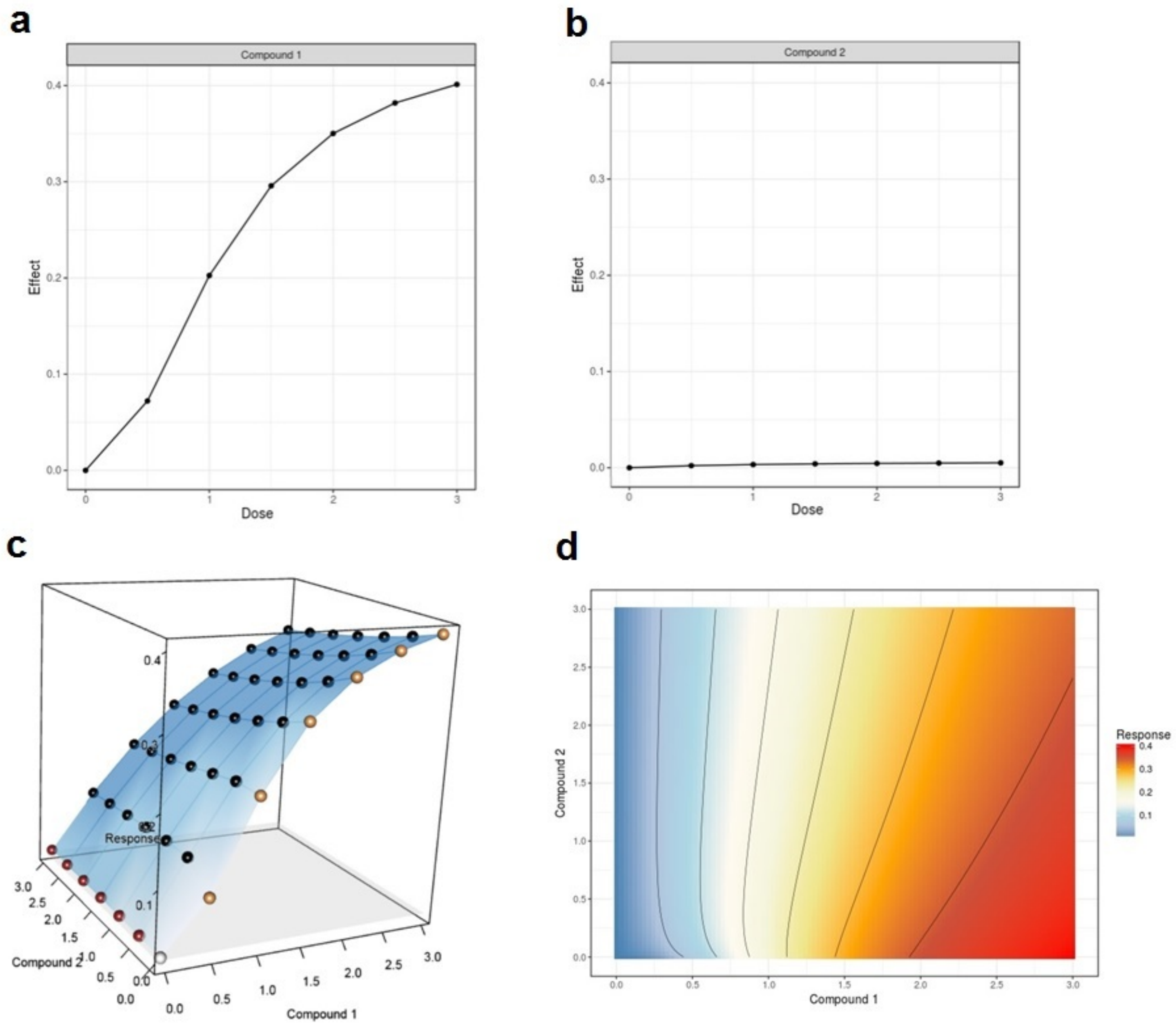


*Figure S4. Example for partial Agonist-neutral Antagonist combination as obtained by R Shiny app. a. dose response curve partial Agonist b. dose response curve neutral Antagonist c. expected BIGL null response surface d. isobologram of the null model.*

## 4 Heatmap O'Neil paper

Heatmaps were generated, similarly to the ones in the original paper, illustrating the number of cell lines with synergy (Figure S5) and antagonism (Figure S6) for each pairwise combination. We used the BIGL model as the null model, while O'Neil used both Bliss independence null model as well as single agent model. However, in general pairs that were found synergistic in the majority of cell lines were also found to be synergistic in our case.

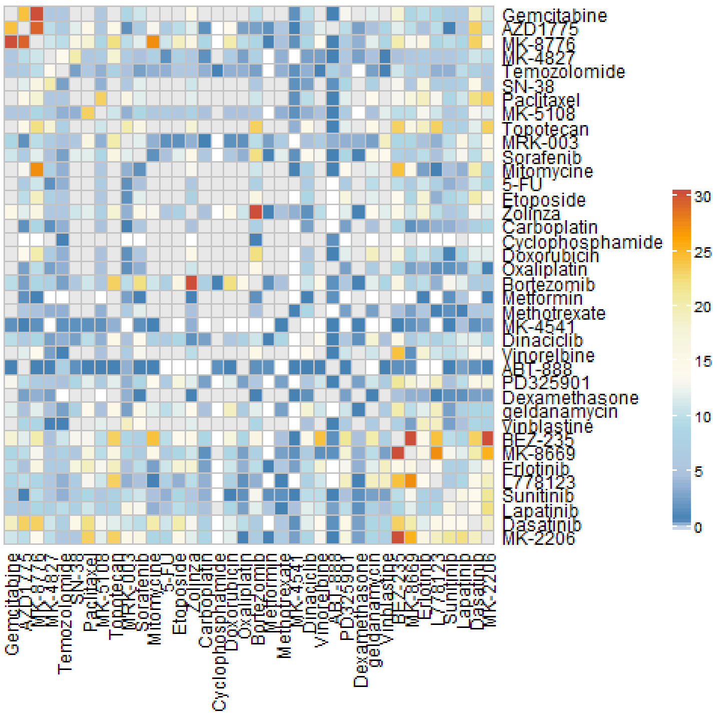


*Figure S5. Heatmap illustrating synergistic combinations. The coloring indicates the number of times a particular pair was called synergistic across the different cell lines. Grey wells are pairs that were not present in the dataset.*

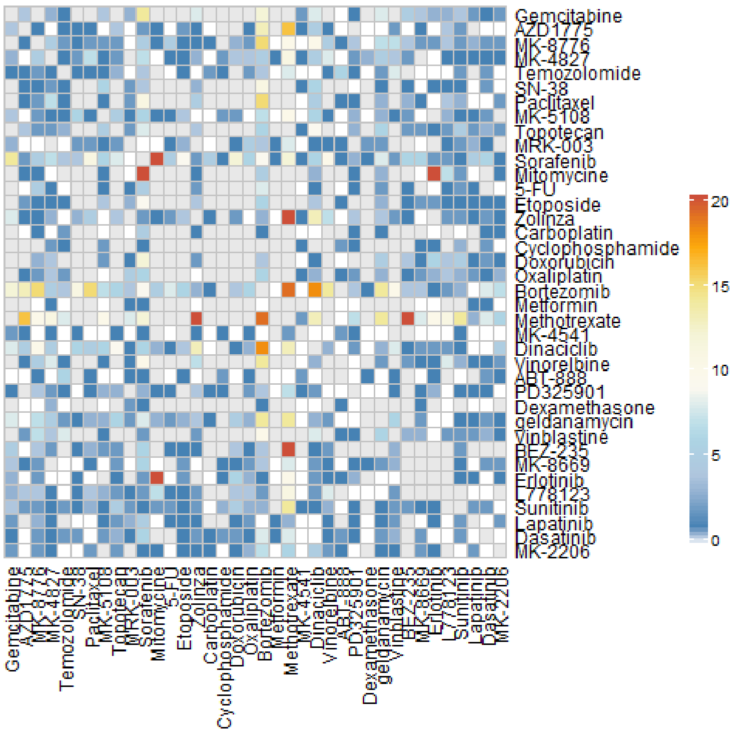


*Figure S6. Heatmap illustrating antagonistic combinations. The coloring indicates the number of times a particular pair was called antagonistic across the different cell lines. Grey wells are pairs that were not present in the dataset.*
